# Regenerative therapy for spinal cord injury using iPSC technology

**DOI:** 10.1186/s41232-020-00149-0

**Published:** 2020-10-16

**Authors:** Narihito Nagoshi, Hideyuki Okano, Masaya Nakamura

**Affiliations:** 1 Department of Orthopaedic Surgery, Keio University School of Medicine, 35 Shinanomachi, Shinjuku-ku, Tokyo, 160-8582 Japan; 2 Department of Physiology, Keio University School of Medicine, 35 Shinanomachi, Shinjuku-ku, Tokyo, 160-8582 Japan

## Abstract

Spinal cord injury (SCI) is a devastating event that causes permanent neurologic impairments. Cell transplantation therapy using neural precursor cells (NPCs) is a promising intervention aiming to replace damaged neural tissue and restore certain functions. Because the protocol to produce human induced pluripotent stem cells (iPSCs) was first established, we have attempted to apply this technology for regenerative therapy in SCI. Our group reported beneficial effects of iPSC-derived NPC transplantation and addressed safety issues on tumorigenicity after grafting. These findings will soon be tested at the clinical trial stage, the protocol of which has already been approved by the Ministry of Health, Labour and Welfare in Japan. Current transplantation therapies treat patients at the subacute phase after injury, highlighting the need for effective treatments for chronic SCI. We recently demonstrated the modest efficacy of gamma secretase inhibitor treatment of iPSC-NPCs before transplantation at the chronic phase. However, more comprehensive strategies involving combinatory therapies are essential to enhance current spinal cord regeneration treatments.

## Background

Spinal cord injury (SCI) occurs when spinal stability breaks down due to high-energy injuries sustained from events such as vehicular or contact sport accidents. The incidence of cervical SCI among the elderly has increased in recent years because of minor traumas such as falls. Current treatments for SCI primarily focus on surgery for spinal realignment and subsequent rehabilitation; however, there are no fundamental therapies to reverse damage to the spinal cord yet.

We aimed to develop a treatment for this challenging pathology; thus, we conducted research exploring cell transplantation therapy using neural precursor cells (NPCs). Since the method for producing induced pluripotent stem cells (iPSCs) was first reported [1, 2], we have addressed our research specifically on the application of human iPSCs for regeneration therapy against SCI. Herein, we outline the efficacy and safety of applying human iPSC-NPCs for SCI treatment, which is currently awaiting clinical application.

## Transplantation therapy using iPSC-NPCs

Around 10 years ago, we developed an induction method that could generate NPCs from iPSCs obtained from mice [3]. Because the NPCs produced from murine embryonic fibroblasts showed no undifferentiated cells in proliferated neurospheres in vitro, we transplanted them into injured murine spinal cords [4]. We found the grafted NPCs differentiated into three neural lineages without tumor formation. The differentiated oligodendrocytes enhanced remyelination for host neuronal axons and induced neurite regrowth of 5-HT^+^ serotonergic fibers around the lesion area, which led to locomotor functional recovery.

These results encouraged advancement toward clinical applications using human iPSCs. We transplanted human iPSC-derived NPCs into the rodent SCI model, successfully demonstrating the differentiation of transplanted cells into neurons that initiated synaptic formation with host axons [5]. The grafted NPCs also promoted angiogenesis, neurogenesis, and the preservation of host tissues. In particular, neuronal regeneration was observed at the caudal site of the lesion epicenter, and serotonergic raphespinal tract axons increased at lumbar intumescence. These beneficial mechanisms contributed to improved motor function. Notably, the iPSC-NPCs did not present tumor formation histologically even more than 3 months after transplantation. These results indicate that iPSCs are effective and safe if validated iPSC lines were selected for cell transplantation therapy. To validate the efficacy of human iPSC-NPCs, we induced SCI models of the non-human primate (common marmoset) and subsequently confirmed that transplanted cells predominantly differentiated into neurons without tumorigenicity, promoted axonal regrowth, and preserved entire host tissues, including the myelination region [6]. These positive effects resulted in functional recovery in the animals receiving NPCs.

A mechanism for functional improvement after transplantation is remyelination of the damaged host axons and restoration of saltatory conduction [7, 8]. We succeeded in generating oligodendrocyte-enriched iPSC-NPCs in vitro with the addition of retinoic acid and purmorphamine, which had the properties of caudalization and ventralizaion along the developmental neural axis, respectively [9]. We then investigated the efficacy of the cells after transplantation using rodent models of SCI. The frequency of differentiation profiles of the grafts was 40% in oligodendrocytes, which was a dramatic enhancement compared with 3% oligo-lineage differentiation from the original NPCs [5]. The transplanted cells exerted robust remyelination on the demyelinated host axons and contributed to the significant preservation of the myelination region. Electrophysiological conduction was restored with a shorter latency of motor evoked potential, leading to locomotor recovery. Therefore, we established that transplantation of oligodendrocyte precursor cell-enriched NPCs is an effective regenerative therapeutic method that promotes remyelination of damaged host axons with favorable functional improvement [10].

The effectiveness of cell therapy using human iPSC-NPCs has been also reported in other studies. Lu et al. established human iPSCs from an 86-year-old healthy male and induced the cells into NPCs [11]. They transplanted the NPCs into a cervical hemisection model of rodents and found that the transplanted cells extended a large number of axons to great distances from the lesion site, over the entire length of the spinal cord at 12 weeks after SCI. The elongated axons and host rodent neurons created synaptic formation.

Fujimoto et al. developed a protocol for generating long-term self-renewing neuroepithelial-like stem (NES) cells from human iPSCs, exhibiting continuous expandability and generating mature functional neurons [12]. When grafted into the injured spinal cord of mice, approximately 80% of these human iPS-lt-NES cells differentiated into neurons, and motor functional recovery was confirmed. To clarify the role of differentiated neurons in the injured spinal cord, they administered diphtheria toxins to ablate the grafts and found that the recovered motor function suddenly deteriorated. Therefore, these results indicate that the transplanted NES cell-derived neurons maintain their functional activity and possibly integrate into host neuronal circuits.

Recently, Khazaei et al. developed human iPSC-NPCs that expressed glial cell-derived neurotrophic factor (GDNF) [13]. GDNF played a role in the suppression of Notch activation, which promoted differentiation of NPCs to more neuronal fate. When transplanting the GDNF-expressing iPSC-NPCs into the cervical SCI model of rodents, the survival rates increased and differentiated more neuronal cells. Interestingly, the generated neurons formed synapse along with host tissues, and the GDNF cells created more excitatory connections. This mechanism contributed to enhancing the electric conduction and improving forelimb motor function.

Given the validity from various reports, human iPSC-NPCs are a promising tool to facilitate cell transplantation therapy for SCI toward clinical application.

## Safety of using iPSC-NPCs

Although NPCs derived from iPSCs exhibited beneficial effects if safety-tested lines were selected, the risk of tumorigenicity after transplantation remains a major concern. Even if iPSCs were considered safe when established, they occasionally present tumor-like growth or differentiation resistance when induced into NPCs and subsequently transplanted into the injured spinal cord [14]. To establish a safe cell therapy, it is necessary to introduce a process of removing tumorigenic cells before or even after transplantation, regardless of the types of cell lines used. Hence, we performed several prophylactic studies against tumorigenicity.

### Safety issue for molecular level

The optimal way to prevent tumor formation in the spinal cord is to select the safe and validated iPSC-NPC lines before transplantation. To identify factors that were associated with tumorigenicity, we evaluated and compared gene expression profiles of tumorigenic and non-tumorigenic iPSC-NPCs by comprehensive DNA methylation analyses [15]. The results revealed that several tumor suppressor genes were methylated in the tumorigenic NPC lines and this epigenomic status could promote the tumor formation after the cell transplantation. Interestingly, tumorigenicity was enhanced when the number of passages increased even for the initially safety-validated iPSC-NPCs. The underlying mechanism for this abnormality was the progression of hypermethylation for the tumor suppressor genes. Altogether, the methylation profiles could be included in the criteria to choose safe iPSC-NPCs in an actual clinical setting, and passage numbers should be limited not to generate aberrational transplanting cells.

### Suppression of tumorigenic cells using a notch signal inhibitor

Ideally, in cell therapy using iPSC-NPCs, tumorigenic cells were eliminated in vitro before transplantation. In this regard, we investigated a γ-secretase inhibitor (GSI), which inhibits Notch signaling, which controls the status of undifferentiated NPCs. Inhibition of this signaling promotes NPCs for additional maturation and neuronal differentiation [16]. In our study, tumorigenic human iPSC-NPCs were treated with GSI in vitro, for only 1 day and already, the cells exhibited neuronal differentiation, a reduction in cell proliferation, and suppression of tumor-related gene expression. Upon transplant into the SCI model of NOD/SCID mice, the iPSC-NPCs generated mature neurons around the injury site and did not form tumors at 89 days after transplantation. On the other hand, in non-GSI-treated NPCs, tumor formation was observed with declining motor function. Thus, pretreatment with GSI can eliminate tumor-initiating cells in human iPSC-NPCs or promote differentiation of the cells into mature neuronal cells. These beneficial mechanisms potentially mitigated the safety issue related to tumorigenicity after cell transplantation.

### Detection of tumor-like proliferating cells using positron emission tomography

In some cell lines, iPSC-NPCs with differentiation-resistant properties caused abnormal cell growth after transplantation [14, 16], which makes immediate identification of these unsafe cell lines critical during cell proliferation in the grafted tissues. The remnant undifferentiated NPCs showed a high expression of the 18-kDa translocator protein (TSPO), also known as the peripheral-type benzodiazepine receptor. Because the TSPO-selective radioligand [^18^F] FEDAC is available as a clinically relevant nuclide, we proposed that the remaining NPCs after transplantation could be detected using positron emission tomography (PET) imaging [17]. When non-tumorigenic iPSC-NPCs were transplanted into central nervous system (CNS) tissue, the cells did not show uptake of the [^18^F] FEDAC in PET imaging. However, the NPCs which had proliferative properties presented nuclide uptake 4 weeks after transplantation. Thus, PET imaging with a NPC-specific nuclide is an effective modality in identifying the remnant undifferentiated cells and ensuring safety in future clinical treatments using cell transplantation.

### Transplantation of tumorigenic iPSC-NPCs with integrated suicide genes

Although pretreating NPCs with GSI prior to transplantation and/or utilizing oligogenic cells may reduce the risk of tumorigenesis, completely eradicating the risk in certain iPSC lines is difficult. We have proposed several possible methods for ablating the transplanted cells retrospectively using suicide gene systems [18, 19]. First, induced caspase-9 was transduced into known tumorigenic iPSC-NPCs following transplantation [18]. These cells were transplanted into the spinal cord of mice and observed for several weeks to allow for tumor growth. When the apoptosis inducer (dimerizer) was systemically injected into these mice, the transplanted cells were successfully ablated with only an insignificant loss of motor function. However, this system killed all the transplanted cells, including fully functional neural cells that may have contributed to functional recovery. In order to spare fully differentiated neural cells from ablation, we tried transducing the herpes simplex virus type I thymidine kinase (HSVtk) gene into the same line of tumorigenic cells [19]. HSVtk is known to phosphorylate its prodrug ganciclovir (GCV) which is toxic to immature/proliferative cells such as tumor cells. A similar experiment with mice transplanted with HSVtk-induced iPSC-NPCs resulted in the selective eradication of tumorigenic cells whilst sparing the mature, differentiated neural cells. Improved motor function was also promoted, affirming the role of transplanted cells within the injured host spinal cord. Because the HSVtk/GCV system has already been applied in some clinical trials without any safety problems [20], this technique can, theoretically, be used in the clinical application of our iPSC project for SCI patients.

## Clinical application of iPSC-NPCs

**Fig. 1:**
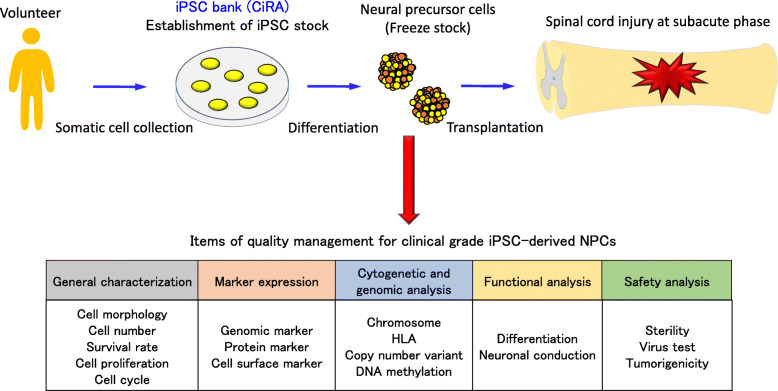
Flow of iPSC-NPC transplantation toward a first-in-man clinical trial

Our efforts toward the establishment of cell therapy using iPSCs will soon attain clinical application [21, 22]. In actual clinical settings, autologous cell transplantation is ideal to prevent immune rejection, but requires the enormous cost of quality assessment for the transplanting cells. This issue changed our direction to allogenic grafts using iPSC stock. The program to develop this stock was launched mainly by the Center for iPS Cell Research and Application (CiRA) at Kyoto University. These iPSCs were generated from human leukocyte antigen (HLA) super-donors who are homozygous at the three major HLA gene loci to maintain a pool of safe iPSC clones corresponding to various HLA types. Consequently, in collaboration with the CiRA, we developed a method to induce NPCs from the clinical-grade human iPSCs (Fig. 1). The established iPSC-NPCs were thoroughly investigated with detailed subitems of quality management, i.e., general characterization, marker expression, and genomic, functional, and safety analyses [23]. Our plan for this trial was approved by the Ministry of Health, Labour and Welfare in Japan in 2019. Patients with American Spinal Injury Association impairment classification A are the target subjects for our clinical study, and the iPSC-NPCs are to be transplanted at 2 to 4 weeks after SCI. The patients will take immunosuppressants for a certain period and will be followed up for 1 year with the proper neurologic evaluations. In the future, validation of iPSC-NPC transplantation at the subacute phase will expand toward graft indication for chronic SCI.

## Cell therapy for chronic SCI

Most studies for SCI have targeted the acute to subacute injury phases for therapeutic interventions because of neural plasticity and reactivity at this stage. However, more than 90% of patients still suffer from their impairments and disabilities at the chronic phase. At this stage, the pathological status presents cavity formation surrounded by glial scar tissue and suppression of neurite extension by myelin-related inhibitory molecules. These chronic phase conditions generate an unfavorable environment for neural cell survival, resulting in failure in functional restoration.

Despite this complicated pathology, we have reported modest but significant functional recovery using GSI for transplanting iPSC-NPCs [24]. We transplanted GSI-treated iPSC-NPCs into the spinal cord of mice 6 weeks after injury and found that the grafted cells predominantly differentiated into mature neuronal cells, whereas undifferentiated, immature NPCs were reduced 12 weeks after transplantation. The differentiated neuronal axons formed inhibitory synaptic connections with host tissues, indicating that suppression of spasticity by the transplanted cells contributed to improved motor coordination. Interestingly, the inhibition of Notch signaling by GSI resulted in the enhanced phosphorylation of p38 mitogen-activated protein kinases, which play an important role in axonal regeneration. Thus, GSI treatment for NPCs has promising favorable outcomes for functional recovery even in the chronic phase. However, the degree of recovery was limited even with GSI administration. Therefore, combination therapy, such as additional rehabilitation, should be taken into consideration to promote spinal cord regeneration.

We previously evaluated the effectiveness of NPC transplantation for chronic SCI combined with rehabilitation therapy using mouse CNS-derived NPCs [25]. The cells were transplanted 49 days after SCI, and the recipients were asked to perform treadmill training for 8 weeks. This combinatory therapy promoted neuronal differentiation in the grafted cells, increased serotonergic neuronal and axonal regeneration, and enhanced coordination and gait. Moreover, these interventions ameliorated sensory abnormalities such as thermal allodynia and tactile hyperalgesia [26]. Therefore, treadmill exercise with NPC transplantation promoted neuronal differentiation and the regeneration and maturation of neural circuits and enhanced the recovery of motor and sensory functions even when intervened at the chronic phase.

## Conclusions

Regenerative therapy using iPSCs is approaching the clinical trial stage. Nevertheless, several issues regarding safety and efficacy remain to be solved. Clinical and fundamental research, as well as mutual feedback, is still necessary in developing more promising treatments, especially in chronic SCI.

## Data Availability

Not applicable.

## Abbreviations

SCI: Spinal cord injury
NPCs: Neural precursor cells
iPSCs: Induced pluripotent stem cells
5-HT: 5-Hydroxytryptamine
GSI: γ-Secretase inhibitor
NOD/SCID: Nonobese diabetic-severe combined immune deficient
TSPO: 18 kDa translocator protein
[^18^F]FEDAC: N-benzyl-N-methyl-2-[7,8-dihydro-7-(2-^18^F-fluoroethyl)-8-oxo-2-phenyl-9H-purin-9-yl]acetamide
PET: Positron emission tomography
CNS: Central nervous system
HSVtk: Herpes simplex virus type I thymidine kinase
GCV: Ganciclovir
CiRA: Center for iPS Cell Research and Application
HLA: Human leukocyte antigen
